# Guided Internet-Based Cognitive Behavioral Therapy for Women With Bulimia Nervosa: Protocol for a Multicenter Randomized Controlled Trial

**DOI:** 10.2196/49828

**Published:** 2023-09-19

**Authors:** Sayo Hamatani, Kazuki Matsumoto, Gerhard Andersson, Yukiko Tomioka, Shusuke Numata, Rio Kamashita, Atsushi Sekiguchi, Yasuhiro Sato, Shin Fukudo, Natsuki Sasaki, Masayuki Nakamura, Ryoko Otani, Ryoichi Sakuta, Yoshiyuki Hirano, Hirotaka Kosaka, Yoshifumi Mizuno

**Affiliations:** 1 Research Center for Child Mental Development University of Fukui Fukui Japan; 2 Division of Developmental Higher Brain Functions United Graduate School of Child Development University of Fukui Fukui Japan; 3 Department of Child and Adolescent Psychological Medicine University of Fukui Hospital Fukui Japan; 4 Division of Clinical Psychology Kagoshima University Hospital Kagishima Japan; 5 Department of Behavioural Sciences and Learning Linköping University Linköping Sweden; 6 Department of Biomedical and Clinical Science Linköping University Linköping Sweden; 7 Department of Clinical Neuroscience Karolinska Institute Stockholm Sweden; 8 Department of Psychiatry Graduate School of Biomedical Sciences Tokushima University Tokushima Japan; 9 Research Center for Child Mental Development Chiba University Chiba Japan; 10 Department of Behavioral Medicine National Institute of Mental Health National Center of Neurology and Psychiatry Tokyo Japan; 11 Department of Psychosomatic Medicine Tohoku University Hospital Sendai Japan; 12 Department of Psychiatry Kagoshima University Graduate School of Medical and Dental Sciences Kagoshima Japan; 13 Child Development and Psychosomatic Medicine Center Dokkyo Medical University Saitama Medical Center Saitama Japan; 14 Department of Neuropsychiatry University of Fukui Fukui Japan

**Keywords:** bulimia nervosa, internet-based cognitive behavioral therapy, ICBT, randomized controlled trial, RCT, protocol, randomized, controlled trial, bulimia, eating, cognitive behavioral therapy, CBT, binge eating, purging, mobile phone

## Abstract

**Background:**

Individual face-to-face cognitive behavioral therapy is known to be effective for bulimia nervosa (BN). Since foods vary considerably between regions and cultures in which patients live, cultural adaptation of the treatment program is particularly important in cognitive behavioral therapy for BN. Recently, an internet-based cognitive behavioral therapy (ICBT) program was developed for Japanese women with BN, adapted to the Japanese food culture. However, no previous randomized controlled trial has examined the effectiveness of ICBT.

**Objective:**

This paper presents a research protocol for strategies to examine the effects of guided ICBT.

**Methods:**

This study is designed as a multicenter, prospective, assessor-blinded randomized controlled trial. The treatment groups will be divided into treatment as usual (TAU) alone as the control group and ICBT combined with TAU as the intervention group. The primary outcome is the total of binge eating and purging behaviors assessed before and after treatment by an independent assessor. Secondary outcomes will include measures of eating disorder severity, depression, anxiety, quality of life, treatment satisfaction, and working alliances. Treatment satisfaction and working alliances will be measured post assessment only. Other measures will be assessed at baseline, post intervention, and follow-up, and the outcomes will be analyzed on an intention-to-treat basis.

**Results:**

This study will be conducted at 7 different medical institutions in Japan from August 2022 to October 2026. Recruitment of participants began on August 19, 2022, and recruitment is scheduled to continue until July 2024. The first participants were registered on September 8, 2022.

**Conclusions:**

This is the first multicenter randomized controlled trial in Japan comparing the effectiveness of ICBT and TAU in patients with BN.

**Trial Registration:**

University Hospital Medical Information Network UMIN000048732; https://center6.umin.ac.jp/cgi-open-bin/ctr_e/ctr_view.cgi?recptno=R000055522

**International Registered Report Identifier (IRRID):**

DERR1-10.2196/49828

## Introduction

### Background

Bulimia nervosa (BN) is an eating disorder characterized by binge eating, purging or fasting, and concerns associated with body shape and weight [1]. Patients with BN often have a distorted body image despite being at standard levels with regard to shape and weight [2]. Consequently, they perform frequent purging or fasting or excessive exercise on the basis of a subjectively abnormal perception of their body shape and weight [3]. Body image distortion involves perceptual disturbances and body dissatisfaction [4]. Repeated episodes of binge eating and inappropriate compensatory behaviors can cause serious physical and mental health concerns. Vomiting after overeating may result in thickened skin on the knuckles, broken teeth, and thyroid dysfunction due to problems with metabolic rate and caloric intake [5,6]. In terms of mental health, patients with BN frequently show low self-esteem, self-harming behaviors, suicidal tendencies, depression, anxiety disorders, sleep disorders, and fatigue or exhaustion [7-12].

### Prevalence of BN and Accessibility to the Treatment

Epidemiological studies confirm that eating disorders are highly prevalent, particularly among women: 2.58% of women in Western countries [13]. The overall observed prevalence of eating disorders was 3.5% between 2000 and 2006 and increased to 7.8% between 2013 and 2018 [14]. A recent meta-analysis estimated that the lifetime and 12-month prevalence of eating disorders was 0.91% and 0.43%, respectively, while the lifetime prevalence of BN was 0.63% [13]. Face-to-face cognitive behavioral therapy (CBT) has been shown to be effective for eating disorders [15], but poor access to treatment remains a problem [16]. In fact, only 19%-36% of people with eating disorders have access to treatment within 1 year [17-19], and 35%-40% of these patients receive standard treatment for eating disorders [20,21]. Furthermore, treatment for eating disorders is sought an average of 5-15 years after the onset of the disorder [16,22]. Access to these appropriate treatments is often hampered by physical barriers, lack of practitioners, and stigma. These problems are particularly associated with face-to-face delivery. Web-mediated interventions may be effective in initiating appropriate early treatment [23,24]. CBT via the internet can dramatically improve access to treatment, especially in developed countries, in which the internet infrastructure is well-developed and information communication devices are widely used.

### A New CBT Model for BN

Enhanced CBT has been shown to be effective in treating eating disorders, including BN. Enhanced CBT encourages the early establishment of healthy and safe eating habits and facilitates behavioral changes and cognitive modification through psychoeducation and self-monitoring. In addition, accumulating evidence from many clinical studies is being integrated to establish CBT techniques that have shown promise for the treatment of eating disorders. In a network analysis, fear of weight gain was suggested to be central to the psychopathology of BN, and hypersensitivity to physical sensations was shown to bridge BN with anxiety or depression [25]. Based on the evidence, this network meta-analysis argued that focusing on the fear of weight gain during exposure therapy and interventions focused on interoceptive exposure is effective in treating BN. However, several other CBTs may also be effective in treating BN, including attentional bias correction training to correct the direction of excessive attention to the body, weight, and food [26], relaxation, and mindfulness to reduce physiological responses such as anxiety and tension [27], traumatic memory care for experiences of being criticized for one’s body type and appearance [28], and impulse control, such as cue exposure [29]. A therapist’s manual for CBT of BN that systematically summarizes these CBT components has been published by Hamatani and Matsumoto [30]. Our research group recently developed a web-based program based on this CBT therapist’s manual by checking the cultural adaptability in Japan [31].

### Objectives

As mentioned earlier, we developed an internet-based cognitive behavioral therapy (ICBT) program for BN that is adapted to Japanese culture and based on Japanese food culture [30,31]. This paper describes the study protocol for a randomized controlled trial (RCT) involving female patients with BN and is designed to evaluate its clinical effects. We will include an intervention group receiving ICBT in addition to treatment as usual (TAU) and a control group receiving TAU alone.

## Methods

### Study Design

This study is designed as a multicenter, prospective, randomized, assessor-blinded clinical trial. The RCT and follow-up study are planned from August 2022 to October 2026 (UMIN000048732). It will be conducted at the following 7 institutions (6 university hospitals and a national medical center) in Japan: University of Fukui Hospital, Kagoshima University Hospital, Chiba University Hospital, Tokushima University Hospital, Dokkyo Medical University, Tohoku University Hospital, and National Center for Neurology and Psychiatry.

### Participants and Eligibility Criteria

Eligible participants will be women aged 13-65 years (1) diagnosed with BN according to the Diagnostic and Statistical Manual of Mental Disorders criteria during a clinical interview [1]; (2) having a BMI over 17.5 kg/m^2^; (3) using computers, tablets, smartphones, etc, on a daily basis, with access to the internet and the minimum necessary information and communications technology skills; and (4) with no history of CBT in the last 2 years. Exclusion criteria include (1) serious mental disorders such as organic brain disorders, psychotic disorders, and drug dependence; (2) imminent risk of suicide; (3) repeated engagement in antisocial behavior; (4) serious progressive physical disease; and (5) difficulty in exposure to feared objects due to severe stress reactions or dissociation symptoms due to acute stress disorder or posttraumatic stress disorder. The purpose of the study is to investigate the effects of an ICBT program that has been adapted based on the culture of Japanese women. Men and women differ in their caloric needs, and most studies regarding eating disorders include female patients. This intervention program is based on the findings of previous studies; therefore, only women will be recruited. In addition, information and communication technology literacy varies considerably by patient age in Japan, with older adults having lower information and communication technology literacy. The intervention in this study is mainly self-help CBT using a web-based platform. Therefore, patients aged 65 years and older will be excluded from the study.

### Recruitment

From August 2022 to July 2024, we will recruit 60 out-participants with female BN through posters, flyers, web or app advertisements (Google Ads, Twitter, and Facebook), newspaper advertisements, etc, posted at medical institutions throughout Japan. Since all participants will continue to be treated, they will be required to obtain clearance from their primary care physician prior to their study enrollment. The participants pay for TAU, but guided ICBT is free. Participants will be rewarded US $82.51 after intervention or waiting completion, regardless of group allocation. These include the amount of time patients spent on the trial and the difficulty of recruiting patients. To participate in this study, some hospitals will require referrals, which may cost the participant money.

### Procedure

**Figure 1:**
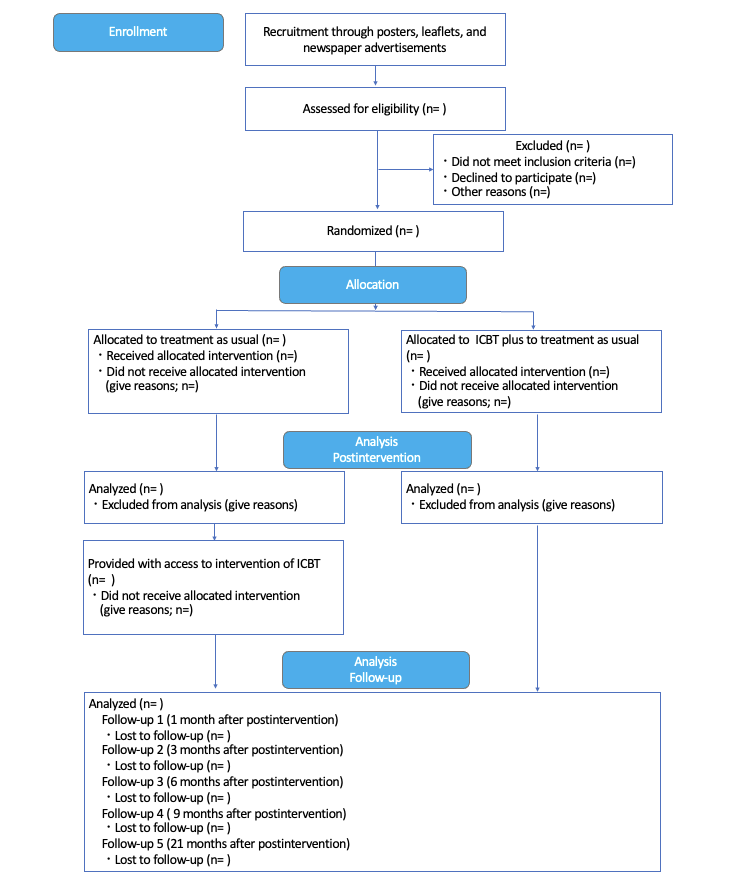
Flowchart of the recruitment and assessments during this study. ICBT: internet-based cognitive behavioral therapy.

Participants will be directed to a website for this study by the various media mentioned earlier. There they will access the study website to apply for enrollment in the study. The clinical trial office at the University of Fukui that receives this application will coordinate the informed consent and eligibility test. Written informed consent will be solicited after the participants are provided research briefing either face-to-face or in a video call. Similarly, in the case of a minor, written informed consent from a legal representative is required. Next, they will complete a screening survey and a structured interview using Mini International Neuropsychiatric Interview version 5 [32,33]. Finally, they will be asked to complete a baseline data questionnaire on the web. The decision regarding the participant’s exclusion or inclusion in the study will be made after they have completed the questionnaire. See Figure 1 for the flow of participants in this RCT.

### Interventions

#### Guided ICBT Program

This ICBT program is based on the treatment manual for BN in Japanese [30]. The ICBT program consists of 1 assessment module and 12 treatment modules, each dedicated to one theme, along with reporting of the development process [31]. Each module includes psychoeducation and practice exercises on CBTs. Each module begins with a video containing complex supporting explanations and messages of encouragement (see Table 1 for details). A secure web-based platform [34] will be used for communication between therapists and participants, distribution of program materials, and collection of assessments. At the start of the intervention, participants will receive an email containing their username and a link to create their own password so that they can log into the platform. Participants will be provided access to 1 new module each week for the duration of the 12-week intervention, starting with module 1. For the first week, an assessment module (module 0) will be provided together with module 1. Once accessible, the modules will be available throughout the treatment duration. Participants who have not viewed the material or practiced it for a particular week will be sent a reminder once a week. If they have any questions, they can contact their therapists via the secure messaging feature on the platform. The therapist is a well-trained and experienced cognitive behavioral therapist (SH). The therapist will receive supervision from KM on the treatment of BN.

**Table 1:** The internet-based cognitive behavioral therapy modules in the randomized controlled trial.

	Module	Explanation
0	Assessment and goal-setting	First, a description of how to use the program and how the program is structured. Data on the user’s basic clinical background, family composition, and chief complaint are collected. Then, treatment goals are set at the end.
1	Psychoeducation and cognitive behavioral model	An overview of the DSM-5^a^ diagnostic classification [1] is provided and the epidemiological features of BN^b^ [35]. Psychoeducation about CBT^c^ for BN is introduced. Perceptions, attention, images, emotions, memories, thoughts, and the vicious circles of habitual behaviors are created graphically [30].
2	Relaxation and mindfulness medication	An introduction to the autonomic nervous system and training in breathing techniques, progressive muscle relaxation, and mindfulness meditation are provided [27,29].
3	Metacognitive training	Metacognition, one of the cognitive functions, is vulnerable in eating disorders [36], and there is evidence that cognitive weakness affects QOL^d^ [37]. Metacognitive interventions may improve eating disorder severity, depression, and QOL [24,38]. This involves dichotomous thinking, emotional judgment, jumping to conclusions, and perfectionism, among others [30,31].
4	Attention bias modification and modification of interpretation for appearance	People with eating disorders tend to focus on body shape, weight, and food overload [39,40]. Exercises are provided that shift attention from food stimuli to neutral stimuli [41,42]. People with eating disorders frequently rate their body shape and weight negatively [43]. When recognizing the threatening bias of one’s natural body shape and weight, the client can use techniques of relaxation and metacognition to handle concerns about perceived body shape and weight.
5	Behavioral experiment for binge eating	Behavioral experiments will be used to see that eating does not lead to uncontrolled weight gain [44].
6	Management of healthy food contents and eating habits	Self-monitoring using food diaries reduces the frequency of binge eating in web-based programs [45]. We propose a Japanese food menu to achieve a well-balanced diet [31]. This approach was developed based on a nutritional rehabilitation called “mechanical diet” by Garner et al [46].
7	Creating an anxiety hierarchy chart and stepwise exposure	Addressing fears associated with overeating can reduce overeating in BN [47,48].
8	Exposure to cues preceding binge eating and purging	Triggers for overeating and purging vary from person to person; however, exposure to such cues can reduce overeating and purging [29].
9	Cognitive restructuring	Negative self-statements associated with eating disorders are frequently observed in BN [49]. Such self-assertions shape the person’s own identity and values [50]. Cognitive restructuring fosters alternative ways of thinking about these self-statements (adaptive thinking).
10	Rewriting of traumatic memory for image of the body	There are often individuals with BN who have negative childhood experiences [51]. If the interpretation of a traumatic memory is tormenting the patient, it should be rewritten to make it safer.
11	Schema work	Rescripting dysfunctional beliefs (schemas) may reduce the frequency of binge eating and purging as well as body shape concerns [52]. Dysfunctional beliefs are organized and alternative beliefs are written down.
12	Prevention of relapse	A summary of what participants have learned from the treatment will be solicited and used to plan future efforts to further reduce symptoms and prevent relapse.

^a^DSM-5: Diagnostic and Statistical Manual of Mental Disorders, Fifth Edition.

^b^BN: bulimia nervosa.

^c^CBT: cognitive behavioral therapy.

^d^QOL: quality of life.

#### TAU (Control Group)

Participants will be allowed to continue receiving counseling, psychotropics, and other medications for the duration of the study. No new drug therapy or additional changes will be recommended during the study period by the study staff. However, the participant’s primary care physician will not be prevented from modifying medications and refer participants to counseling or second-line therapy if deemed clinically appropriate. All changes in TAU will be documented along with the reasons for these changes. Participants in the control group (TAU) will be offered ICBT after a waiting period as well.

### Measures

#### Demographic Data

Participants will be asked about age, educational level, marital status, employment status, BMI, age at BN onset, duration of BN, comorbidities, and the presence or absence of psychotropic or antidepressant drug use.

#### Primary Outcome

The primary outcome will be the weekly combined frequency of binge eating and purging behaviors. These will be measured by an independent assessor whose allocations are masked.

#### Secondary Outcomes

Secondary self-reported outcomes will be the Eating Disorders Examination Questionnaire [53,54], Patient Health Questionnaire-9 [55,56], Generalized Anxiety Disorder Scale-7 [56,57], EQ-5D [58,59], Brunnsviken Brief Quality of Life Scale [60] at baseline, postintervention, and follow-up (1-, 3-, 6-, 9-, and 21-month follow-ups). Assessments based on the Working Alliance Inventory-Short Form [61] and Client Satisfaction Questionnaire [62,63] will be performed only after the treatment.

### Sample Size

The required sample size was calculated using statistical analysis software (G*Power, version 3.1; Heinrich-Heine-Universitaet Duesseldorf). An effect size of Cohen *d*=0.90 from a previous study was used as a reference [64]. The significance level was set at *P* values <.05 for 2-sided tests. The power was set to 80%. The required sample size (assigned number of participants) will be 60 when considering a 30% noncompletion rate based on a previous study [65]. Completers in this study are defined as those who complete at least 80% of the module [65]. In addition, dropouts in this study are defined as those who drop out by not providing postevaluation data or those who discontinue their participation due to adverse events.

### Randomization

Participants confirmed to be eligible and enrolled will be randomized in a 1:1 ratio to either the intervention (TAU added guided ICBT) or TAU alone groups by the truncated binomial design using UMIN’s (University Hospital Medical Information Network) computer program (UMIN Center). The truncated binomial design is that complete randomization will be performed until the number of randomizations to any group reaches n/2, after which all subjects are allocated to the other group to prevent an imbalance in the number of people.

### Statistical Analysis Plan

Statistical analysis will be performed using SPSS Statistics software (version 29; IBM Corp). Statistical analysis will be performed in accordance with the CONSORT (Consolidated Standards of Reporting Trials) guidelines and based on the intention-to-treat principle (Multimedia Appendix 1). Missing data will be handled by multiple imputations. Unpaired *t* test and Fisher exact test will be used to investigate the difference between the 2 groups at baseline. To compare treatment effects, we will use analysis of covariance for primary and all secondary outcomes. As a covariate, we plan to include a scale that is significantly different between the 2 groups at baseline. We also will perform a per-protocol analysis by excluding patients who deviate from the protocol. Analysis of covariance will also be performed on the follow-up data. However, if there are many missing values, we would consider generalized linear mixed models. All *P* values are 2-tailed, and *P* values <.05 will be considered statistically significant.

### Ethical Considerations

The clinical trial protocol was approved by the Research Ethics Committee of the University of Fukui on August 15, 2022 (20220054), and was registered in the UMIN Domestic Clinical Trial Database (UMIN000048732). Each participant will then be informed that all participants will receive TAU from the general practitioner and that half of the participants will receive ICBT in addition to TAU. All adverse events will be reported, and serious adverse events will be reported immediately to the institutional review board. An adverse event is defined as any symptom or illness occurring during a clinical trial, whether related to the ICBT program or not. The results of the trial will be published in the appropriate journal, regardless of the outcomes. The trial will be implemented and reported in accordance with the CONSORT recommendations.

## Results

Recruitment began on August 19, 2022, following the attainment of ethics approval on August 15, 2022, and the granting of permission to conduct the study on August 18, 2022. The first participant was enrolled on September 8, 2022, and recruitment is scheduled until July 2024. First, we plan to report the results using pre-post data from the RCT design. Subsequently, we will also report the 2-year long-term effects of those who received ICBT.

## Discussion

### Anticipated Findings

This paper presents the research protocol for the first RCT designed to investigate the effectiveness of therapist-guided ICBT for female Japanese patients with BN. Notably, a recent RCT demonstrated the efficacy of ICBT for binge eating disorder and other specified feeding or eating disorders [66]. However, although that study, conducted in the Netherlands, showed promising results for guided ICBT in these eating disorders, it involved participants with varying BMI of 19.5 or 40.0 kg/m^2^ and did not include patients specifically diagnosed with BN. In contrast, this study protocol uses specific eligibility criteria, focusing solely on individuals with BN as the primary diagnosis while excluding other eating disorders, such as binge eating disorder. Consequently, the patient cohort to be included in our RCT is anticipated to be homogenous, thereby bolstering confidence in the treatment’s effectiveness. Meanwhile, a systematic review and meta-analysis examining e-therapy’s impact on eating disorders reported inconclusive evidence regarding the effect on overeating frequency in patients with BN [67]. Although this meta-analysis found improvements in various aspects, such as binge eating, vomiting, and laxative misuse, and in the discontinuation rate of overeating symptoms at the intervention’s conclusion, the estimates were imprecise due to the heterogeneity in the quality of studies included in the analysis. Therefore, the authors concluded that e-therapy, including guided ICBT, could be effective for the management of eating disorders; however, further research is needed. A recent single-arm ICBT study targeting patients with eating disorders, including BN, reported positive outcomes [65]. In evidence-based medicine, individual RCT holds the highest level of evidence among single clinical trials [68]. Nevertheless, the outcomes of single-arm clinical trials lacking a control group should be interpreted with caution. In this context, our RCT may expand the understanding of the efficacy of guided ICBT for BN. To our knowledge, even recent RCTs have not provided effect estimates for patients with BN alone, as they also included other eating disorders [69,70]. Therefore, this study, by adhering closely to the RCT protocol described herein, will be well-positioned to offer valuable insights into the efficacy of guided ICBT on the frequency of overeating and purging along with any secondary symptoms in patients with BN. Furthermore, to the best of our knowledge, this is the first study designed to examine the effects of ICBT on behavioral changes related to eating styles in Japan. This is especially significant given Japan’s distinct food culture and medical system that sets it apart from Western countries. The investigation into the effects of ICBT for BN within the context of Japan carries particular importance, as it navigates cultural nuances and differences in the medical system. Therefore, a multicenter study design is more likely to reduce bias and yield findings with broader generalizability.

### Limitation

One limitation of the study is that it would not allow elucidation of the specific effects of the ICBT program because it does not use a psychological placebo group to control for nonspecific factors.

### Conclusions

This RCT aims to validate and prove evidence for the efficacy of a Japanese culture–adapted ICBT program for female patients with BN. This will allow discussion of efficacy beyond the preliminary results obtained to date. The low availability of CBT is an international problem, and the implementation rate of CBT in Japanese psychiatric clinics is extremely low at 6.2% [71]. If positive results are obtained from this RCT, more patients with BN could receive early treatment, which could lead to early improvement in eating disorders that tend to be prolonged and chronic.

## Appendix

Multimedia Appendix 1 CONSORT-eHEALTH checklist.

## Abbreviations

BN: bulimia nervosa
CBT: cognitive behavioral therapy
CONSORT: Consolidated Standards of Reporting Trials
ICBT: internet-based cognitive behavioral therapy
RCT: randomized controlled trial
TAU: treatment as usual
UMIN: University Hospital Medical Information Network

## Data Availability

Data sharing is not applicable to this paper as no data sets were generated or analyzed during this study.
